# Nutritional Status Association With Sarcopenia in Patients Undergoing Maintenance Hemodialysis Assessed by Nutritional Risk Index

**DOI:** 10.3389/fnut.2022.896427

**Published:** 2022-05-13

**Authors:** Masafumi Kurajoh, Katsuhito Mori, Mizuki Miyabe, Shota Matsufuji, Mitsuru Ichii, Tomoaki Morioka, Akane Kizu, Yoshihiro Tsujimoto, Masanori Emoto

**Affiliations:** ^1^Department of Metabolism, Endocrinology, and Molecular Medicine, Graduate School of Medicine, Osaka Metropolitan University, Osaka, Japan; ^2^Department of Nephrology, Graduate School of Medicine, Osaka Metropolitan University, Osaka, Japan; ^3^Division of Internal Medicine, Dialysis Center, Inoue Hospital, Osaka, Japan; ^4^Division of Rehabilitation, Inoue Hospital, Osaka, Japan

**Keywords:** nutritional status, nutritional risk index (NRI), sarcopenia, AWGS 2019, hemodialysis

## Abstract

**Background:**

Malnutrition and sarcopenia are frequently observed in patients undergoing maintenance hemodialysis (MHD). To elucidate whether malnutrition is associated with sarcopenia in those cases, the relationship of nutritional status with sarcopenia was investigated.

**Methods:**

Nutritional status was assessed using a nutritional risk index (NRI) developed for patients undergoing MHD. This retrospective cross-sectional study included 315 MHD patients (199 males, 116 females), who were divided into low-risk (score 0–7) and medium-/high-risk (score 8–13) groups. Sarcopenia and severe sarcopenia, along with low muscle mass, low muscle strength, and low physical performance were defined using the Asian Working Group for Sarcopenia 2019 criteria.

**Results:**

The median NRI score was 5.0, while the prevalence of medium-/high-risk cases among the patients was 31.1%. Additionally, the rates of those with low muscle mass, low muscle strength, and low physical performance were 55.9, 60.6, and 31.4%, respectively, while those of sarcopenia and severe sarcopenia were 44.1 and 20.0%, respectively. Multivariable logistic regression analyses revealed a significant (*P* < 0.001) association of NRI score with sarcopenia [odds ratio (OR) 1.255, 95% confidence interval (CI) 1.143–1.377] and severe sarcopenia (OR 1.257, 95% CI 1.122–1.407), as well as low muscle mass (OR 1.260, 95% CI 1.157–1.374), low muscle strength (OR 1.310, 95% CI 1.178–1.457), and low physical performance (OR 1.216, 95% CI 1.104–1.339). Furthermore, medium-/high-risk status showed a significant (*P* < 0.05) association with sarcopenia (OR 2.960, 95% CI 1.623–5.401) and severe sarcopenia (OR 2.241, 95% CI 1.151–4.362), as well as low muscle mass (OR 2.141, 95% CI 1.219–3.760), low muscle strength (OR 7.665, 95% CI 3.438–17.091), and low physical performance (OR 2.570, 95% CI 1.401–4.716).

**Conclusions:**

These results suggest that malnutrition contributes to sarcopenia/severe sarcopenia in MHD patients by reducing muscle mass and strength, and physical performance.

## Introduction

Protein-energy wasting (PEW), which refers to loss of body protein mass and fuel reserves, is common in patients with end-stage kidney disease (ESKD), and also known to be associated with increased morbidity and mortality (1–3). For diagnosis of PEW, an international society panel of renal nutrition and metabolism experts has recommended use of four primary established categories; serum chemistry, body mass, muscle mass, and dietary intake (1).

Malnutrition is frequently observed in ESKD patients (4, 5) and nutritional screening tools, including the subjective global assessment of nutrition (SGA) (6), modified versions of the SGA (7, 8), and malnutrition–inflammation score (MIS) (9), are suggested to be potential clinical markers of PEW status (1). However, those tools require subjective assessment and judgment by the examiner, which may reduce reproducibility, and are also time-consuming, thus difficult to integrate into routine clinical practice. Recently, based on the concept of PEW, a nutritional risk index (NRI) was developed using data from the Japanese Society for Dialysis Therapy Renal Data Registry to predict the prognosis of maintenance hemodialysis (MHD) patients (10), which was later validated in the Q-Cohort Study that included elderly MHD patients (11). The NRI is comprised of four objective variables routinely measured in clinical settings, and has been shown to be a simple and useful nutritional screening tool for MHD patients in Japan (12).

Sarcopenia, characterized as loss of skeletal muscle mass and function (13), is also frequently observed in MHD patients (14, 15) and that condition is called uremic sarcopenia. Uremic sarcopenia is induced by complex pathways, including uremic changes, inflammation, myocellular changes, hormonal changes, and comorbid conditions (16). In related studies, nutritional intervention methods have been shown capable of increasing lean body mass, arm muscle circumference, or handgrip strength in patients undergoing MHD (17–19), suggesting a close relationship of nutritional status with sarcopenia in those cases.

However, the association of nutritional status with sarcopenia has not been fully clarified in regard to MHD patients. Therefore, the present study aimed to elucidate the relationship of malnutrition with sarcopenia in such patients by investigating the association of nutritional status, assessed by use of the NRI, with sarcopenia, assessed by the Asian Working Group for Sarcopenia (AWGS) 2019 criteria (20).

## Materials and Methods

### Study Design and Participants

This cross-sectional retrospective study was conducted at Inoue Hospital (Osaka, Japan) (21). Among 334 MHD patients who underwent measurements of muscle mass, muscle strength, and physical performance from January 2019 to December 2019, those who had been receiving therapy for <6 months (*n* = 3) or with missing data regarding nutritional status (*n* = 12) were excluded. Furthermore, patients with malignancy, acute illness, infection, or liver cirrhosis (*n* = 4) were further excluded from the present analysis. As a result, 315 patients undergoing MHD (199 males, 116 females) were retrospectively analyzed in the present study.

Body weight refers to “dry weight” in the present study. Body mass index (BMI) was defined as body weight in kilograms divided by the square of height in meters. Diabetes mellitus was defined based on the American Diabetes Association criteria or treatment for diabetes mellitus (22). Past history of cerebrovascular disease was defined as prior occurrence of intracerebral hemorrhage, transient ischemic attack, subarachnoid hemorrhage, and/or ischemic stroke (23, 24).

Owing to the retrospective nature of the investigation, the Inoue Hospital Ethics Committee waived the need/requirement for obtaining informed consent (approval no. 236). All data subjected to analysis were collected from relevant patient medical charts and records, after approval of the study protocol. The present study was conducted in full accordance with the principles of the Declaration of Helsinki.

### Blood Sample Collection

Blood was drawn from each patient with or without fasting before the start of the first MHD session of the week. The level of albumin in serum was analyzed using a bromocresol purple method, while that of the serum standard (low-sensitive) C-reactive protein (CRP) was analyzed using a latex agglutination immunoassay (reference range; 0–4.5 mg/L) at the hospital. Other standard laboratory parameters including levels of total cholesterol, creatinine, and hemoglobin were analyzed by use of a routine laboratory method at the hospital (23, 24).

### Nutritional Risk Index

Nutritional status was assessed using NRI score (10), which was calculated based on creatinine, total cholesterol, serum albumin, and BMI (Table 1).

**Table 1:** NRI score.

**NRI score component**	**Category cut-off point**	**Score**
Low serum creatinine level	Age ≥65; male <9.7 mg/dl, female <8.0 mg/dl	4
	Age <65; male <11.6 mg/dl, female <9.7 mg/dl	
Low serum total cholesterol level	<130 mg/dl	1
High serum total cholesterol level	≥220 mg/dl	2
Low serum albumin level	Age ≥65; <3.5 g/dl	4
	Age <65; <3.7 g/dl	
Low BMI	<20 kg/m^2^	3

*NRI score = low serum creatinine level + abnormal serum total cholesterol level + low serum albumin level + low BMI*.

*NRI, nutritional risk index; BMI, body mass index*.

Based on their NRI score, the enrolled patients were divided into the low-risk (score 0–7) and medium-/high-risk (score 8–13) groups (10).

### Methods for Muscle Mass, Muscle Strength, and Physical Performance Measurements

As a part of routine clinical care for MHD patients at Inoue Hospital, muscle mass, muscle strength, and physical performance are generally measured after the end of MHD treatment at the beginning of the week of their birthday (21). The participants were informed that they were scheduled to be evaluated in regard to muscle mass, muscle strength, and physical performance on a day when undergoing treatment prior to the scheduled test date. Muscle strength and physical performance were measured by 10 different examiners, who received training and standardized their methods in advance.

### Determination of Muscle Mass

Muscle mass was assessed using a whole body dual-energy X-ray absorptiometry system (Horizon A; Hologic Inc, MA, USA) (25). Appendicular skeletal muscle was considered as an extremity fat-free mass lacking bone mineral content. Height-adjusted muscle mass was calculated as appendicular skeletal muscle in kilograms divided by height in meters squared (kg/m^2^) (15).

### Measurement of Muscle Strength

Muscle strength was assessed with a handgrip strength test using a Smedley-type hand dynamometer (T.K.K.5101; Takei, Niigata, Japan), with the examination conducted by an experienced research staff member. The patients were instructed to apply maximum force in an upright position and hold the grip with their arm extended (26, 27). Measurements were repeated two times with both hands, and the maximum value (kg) was recorded.

### Assessment of Physical Performance

A five-time chair stand test was conducted to assess physical performance based on the AWGS 2019 criteria (20). Each patient was instructed to stand and sit five times as fast as possible from a sitting position on a chair with a seat height of 40 cm and straight back without armrests, with their hands on their shoulders and arms crossed across the chest, as previously reported in the CHAIR (change hemodialysis patients' activity and impaired functions by chair stand exercise) study (28). They initiated the five-time chair standing test when an experienced research staff member spoke the word “start,” then the time (seconds) to complete the fifth repetition was recorded (29, 30).

### Definitions of Low Muscle Mass, Low Muscle Strength, and Low Physical Performance

Based on the AWGS 2019 criteria, low muscle mass was defined as height-adjusted muscle mass of <7.0 kg/m^2^ for males and <5.4 kg/m^2^ for females, low muscle strength as handgrip strength of <28 kg for males and <18 kg for females, and low physical performance as a five-time chair stand test time of ≥12 seconds for both males and females (20).

### Diagnosis of Sarcopenia and Severe Sarcopenia

For diagnosis of sarcopenia and severe sarcopenia, the AWGS 2019 criteria were used (20). In brief, subjects with low muscle mass, plus either low muscle strength or low physical performance were diagnosed as having sarcopenia, while those with low muscle mass, plus both low muscle strength and low physical performance were diagnosed as having severe sarcopenia.

### Statistical Analysis

Values are expressed as the percent or median (interquartile range). A chi-squared test (categorical variables) and Mann–Whitney's *U* test (continuous variables) were used to compare variables between groups. Spearman's correlation coefficient was used to determine correlations between variables. Serum CRP values below the detection limit (0.1 mg/L) were handled as 0.1 mg/L. Before performing multivariable regression analysis, the serum level of CRP was logarithmically transformed (log) to follow a normal distribution. Multivariable linear regression analysis was used to determine whether the NRI score was independently associated with muscle mass, muscle strength, or physical performance, following adjustments using known risk factors, including age, gender, hemodialysis duration, presence of diabetes mellitus, history of cerebrovascular disease, use of intravenous and/or oral vitamin D, and hemoglobin and CRP levels. Multivariable logistic regression analyses were performed to determine whether NRI score, or a finding of medium- or high-risk assessed by the NRI was independently associated with presence of low muscle mass, low muscle strength, low physical performance, sarcopenia or severe sarcopenia, after adjustments using the known risk factors noted above. All statistical analyses were performed using the Statistical Package for the Social Sciences software package (IBM SPSS Statistics for Windows, 27.0, IBM Corp., Armonk, NY, USA). All reported *P* values are two-tailed, with a value < 0.05 considered to indicate statistical significance.

## Results

### Clinical Characteristics of Patients

Table 2 presents characteristics of the enrolled patients (*n* = 315). The rates of prevalence of low serum creatinine level, low serum total cholesterol level, high serum total cholesterol level, low serum albumin level, and low BMI based on the NRI score cut-off point were 38.1% (*n* = 120), 21.0% (*n* = 66), 7.3% (*n* = 23), 65.7% (*n* = 207), and 28.6% (*n* = 90), respectively. The median NRI score was 5.0 and the prevalence of patients in the medium-/high-risk group (NRI score ≥8) was 31.1% (*n* = 98). The prevalence ratios for low muscle mass, low muscle strength, and low physical performance were 55.9% (*n* = 176), 60.6% (*n* = 191), and 31.4% (*n* = 99), respectively, while those for sarcopenia and severe sarcopenia were 44.1% (*n* = 139) and 20.0% (*n* = 63), respectively. Finally, as compared to female subjects, male subjects had significantly (*P* < 0.001) higher values for height-adjusted muscle mass and handgrip strength, and significantly (*P* < 0.05) lower values for five-time chair stand test time (Table 2). However, the frequency of sarcopenia and severe sarcopenia was not significantly different between male and female subjects.

**Table 2:** Clinical characteristics of patients (*n* = 315) stratified by gender and nutritional status assessed by NRI.

	**Total (*n* = 315)**	**Male (*n* = 199)**	**Female (*n* = 116)**	***P***	**Medium-/high-risk group (*n* = 98)**	**Low-risk group (*n* = 217)**	***P*** **-Value**
Age, years	69.0 (59.0–76.0)	69.0 (58.0–76.0)	70.0 (61.3–75.8)	0.502	71.0 (60.8–79.0)	68.0 (58.0–75.0)	0.056
Male, *n* (%)	199 (63.2)				69 (70.4)	130 (59.9)	0.074
Duration of hemodialysis, years	6.0 (2.0–16.0)	5.0 (2.0–13.0)	10.0 (5.0–20.0)	<0.001	8.0 (3.0–20.0)	6.0 (2.0–13.0)	0.128
Diabetes mellitus, *n* (%)	100 (31.7)	74 (37.2)	26 (22.4)	0.007	42 (42.9)	58 (26.7)	0.004
Cerebrovascular disease, *n* (%)	53 (16.8)	39 (19.6)	14 (12.1)	0.085	25 (25.5)	28 (12.9)	0.006
Vitamin D user, *n* (%)	261 (82.9)	162 (81.4)	99 (85.3)	0.371	75 (76.5)	186 (85.7)	0.045
Lipid lowering agent user, *n* (%)	85 (27.0)	51 (25.6)	34 (29.3)	0.478	28 (28.6)	57 (26.3)	0.670
Hemoglobin, g/dl	11.2 (10.5–11.9)	11.2 (10.5–11.9)	11.2 (10.5–12.0)	0.654	10.9 (10.3–11.6)	11.4 (10.6–12.2)	0.002
CRP, mg/L	1.2 (0.5–3.6)	1.3 (0.5–4.2)	1.1 (0.4–3.0)	0.167	1.8 (0.7–6.6)	1.0 (0.5–3.3)	0.011
NRI score	5.0 (4.0–8.0)	5.0 (3.0–8.0)	6.0 (4.0–7.8)	0.615	8.0 (9.0–11.0)	4.0 (1.0–5.0)	<0.001
Creatinine, mg/dl	10.0 (8.5–11.5)	10.5 (9.0–12.5)	9.3 (8.2–10.5)	<0.001	8.4 (7.4–9.4)	10.8 (9.6–12.5)	<0.001
Total cholesterol, mg/dl	160.0 (133.0–186.0)	153.0 (125.0–176.0)	175.5 (151.3–198.0)	<0.001	144.5 (122.0–176.3)	168.0 (144.5–187.5)	<0.001
Albumin, g/dl	3.4 (3.2–3.6)	3.5 (3.2–3.7)	3.4 (3.2–3.5)	0.005	3.3 (3.1–3.4)	3.5 (3.3–3.7)	<0.001
BMI, kg/m^2^	21.7 (19.3–24.1)	22.3 (20.0–24.8)	20.8 (18.9–23.1)	0.001	20.9 (18.8–23.1)	22.2 (19.8–24.6)	0.003
Height-adjusted muscle mass, kg/m^2^	6.2 (5.4–7.1)	6.7 (6.1–7.5)	5.4 (5.1–6.0)	<0.001	6.0 (5.3–6.8)	6.3 (5.6–7.3)	0.016
Handgrip strength, kg	22.7 (16.8–28.7)	26.8 (22.1–31.4)	16.6 (13.3–19.2)	<0.001	20.2 (15.2–25.4)	24.2 (17.5–30.2)	<0.001
Five-time chair stand test time, seconds	10.2 (8.2–13.2)	10.0 (8.1–12.5)	10.7 (8.7–14.9)	0.041	11.9 (9.8–15.3)	9.5 (7.6–11.9)	<0.001
Low muscle mass, *n* (%)	176 (55.9)	120 (60.3)	56 (48.3)	0.038	68 (69.4)	108 (49.8)	0.001
Low muscle strength, *n* (%)	191 (60.6)	113 (56.8)	78 (67.2)	0.067	83 (84.7)	108 (49.8)	<0.001
Low physical performance, *n* (%)	99 (31.4)	54 (27.1)	45 (38.8)	0.032	46 (46.9)	53 (24.4)	<0.001
Sarcopenia, *n* (%)	139 (44.1)	95 (47.7)	44 (37.9)	0.091	63 (64.3)	76 (35.0)	<0.001
Severe sarcopenia, *n* (%)	63 (20.0)	39 (19.6)	24 (20.7)	0.815	32 (32.7)	31 (14.3)	<0.001

*Values are expressed as number (%) or median (interquartile range)*.

*P-values indicate comparisons of median values between the groups (Mann–Whitney's U test) or percentages (chi-square test)*.

*NRI, nutritional risk index; CRP, C-reactive protein; BMI, body mass index*.

The primary disease related to renal failure in the enrolled patients was diabetic nephropathy in 91 (28.9%), chronic glomerulonephritis in 86 (27.3%), nephrosclerosis in 38 (12.1%), polycystic kidney disease in 20 (6.3%), graft loss in 17 (5.4%), unclassifiable nephritis in 7 (2.2%), congenital anomaly in the kidneys or urinary tract in 4 (1.3%), kidney or urinary tract tumor in 3 (1.0%), pregnancy-induced hypertension in 3 (1.0%), malignant hypertension in 2 (0.6%), autoimmune nephritis in 2 (0.6%), urinary tract obstruction in 2 (0.6%), chronic pyelonephritis in 1 (0.3%), acute kidney injury in 1 (0.3%), other disease in 4 (1.3%), and unknown etiology in 34 (10.8%).

### Associations of NRI Score With Muscle Mass, Muscle Strength, and Physical Performance

NRI score showed weak to moderate though significant correlation with height-adjusted muscle mass (muscle mass; ρ = −0.359, *P* < 0.001), handgrip strength (muscle strength; ρ = −0.361, *P* < 0.001), and five-time chair stand test time (physical performance; ρ = 0.407, *P* < 0.001). To further examine whether NRI score is independently associated with those factors, multivariable linear regression analyses were performed (Table 3), which showed the score to be significantly associated with muscle mass (β = −0.310, *P* < 0.001), muscle strength (β = −0.200, *P* < 0.001), and physical performance (β = 0.195, *P* = 0.001), while age and gender also showed a significant (*P* < 0.01) association.

**Table 3:** Multivariable linear regression analysis of factors possibly associated with muscle mass, muscle strength, and physical performance.

	**Muscle mass**		**Muscle strength**		**Physical performance**	
	**β**	***P*** **-Value**	**β**	***P*** **-Value**	**β**	***P*** **-Value**
Age	−0.244	<0.001	−0.384	<0.001	0.293	<0.001
Gender (male/female = 1/0)	0.451	<0.001	0.568	<0.001	−0.139	0.009
Duration of hemodialysis	−0.072	0.123	−0.206	<0.001	0.033	0.566
Diabetes mellitus (yes/no = 1/0)	0.129	0.005	−0.037	0.292	0.051	0.360
Cerebrovascular disease (yes/no = 1/0)	−0.015	0.718	−0.044	0.185	0.051	0.329
Vitamin D user (yes/no = 1/0)	−0.010	0.806	0.057	0.082	0.032	0.532
Hemoglobin	0.050	0.238	0.042	0.205	−0.002	0.974
Log CRP	0.087	0.046	−0.026	0.440	0.074	0.173
NRI score	−0.310	<0.001	−0.200	<0.001	0.195	0.001
Adjusted *R*^2^/*P*	0.465/ <0.001		0.674/ <0.001		0.179/ <0.001	

*Standardized partial regression coefficient values (β values) and level of significance are shown*.

*CRP, C-reactive protein; NRI, nutritional risk index*.

### Associations of Nutritional Status With Low Muscle Mass, Low Muscle Strength, and Low Physical Performance

**Figure 1:**
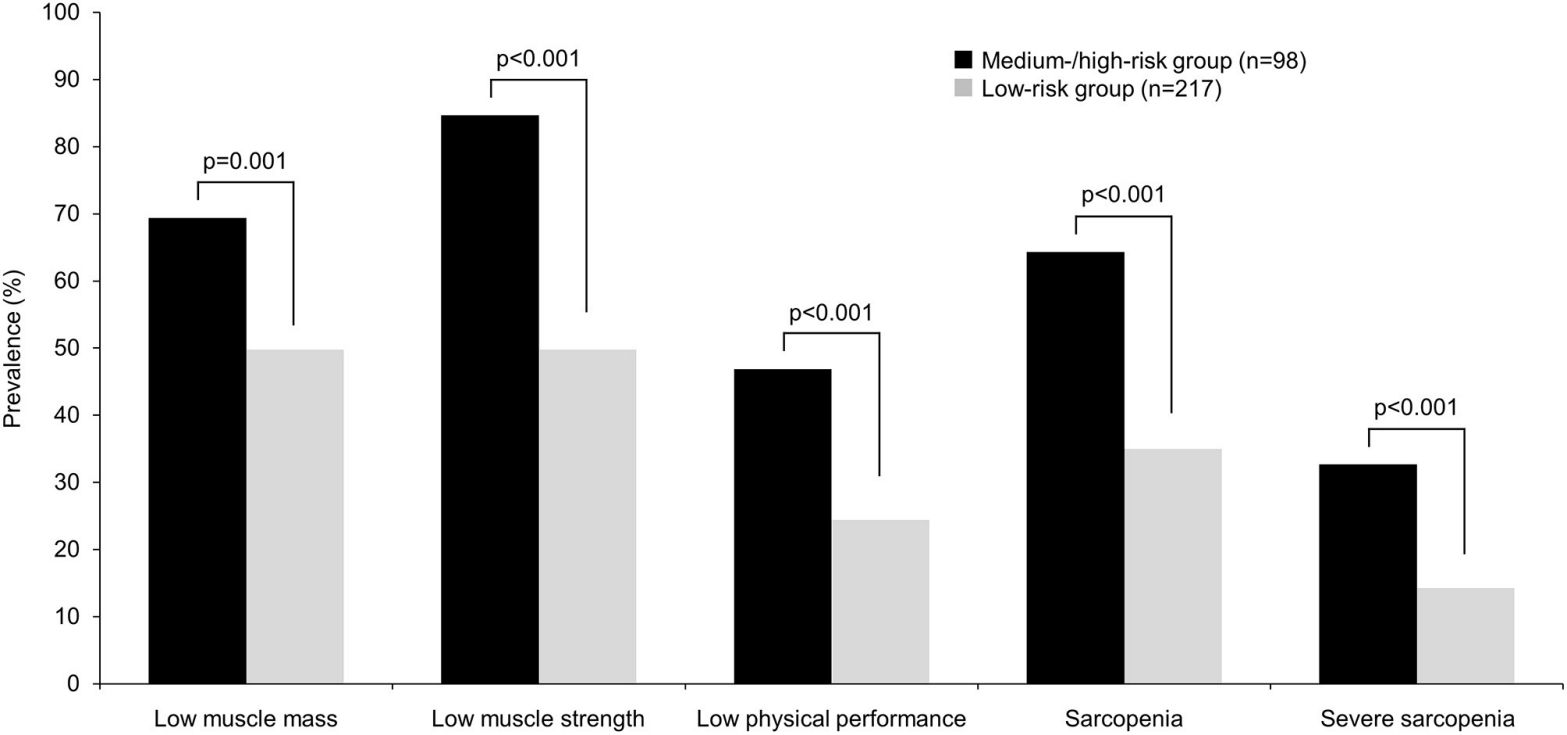
Prevalence of sarcopenia and its components in medium-/high-risk and low-risk groups. Patients were divided into low-risk (score 0–7) and medium-/high-risk (score 8–13) groups based on NRI score. NRI, nutritional risk index.

NRI score was significantly higher in patients with low muscle mass [7.0 (4.0–8.0) vs. 4.0 (1.0–7.0), *P* < 0.001], low muscle strength [7.0 (4.0–8.0) vs. 4.0 (0.3–5.8), *P* < 0.001], and low physical performance [7.0 (5.0–9.0) vs. 4.0 (3.0–7.0), *P* < 0.001], as compared to patients without those factors present. Furthermore, the medium-/high-risk group (*n* = 98) showed a significantly (*P* < 0.01) higher prevalence for low muscle mass, low muscle strength, and low physical performance as compared to the low-risk group (*n* = 217; Table 2, Figure 1). To examine whether NRI score and medium-/high-risk were independently associated with those factors in the patient cohort, multivariable logistic regression analyses were performed (Table 4). The results showed that NRI score was significantly associated with low muscle mass [odds ratio (OR) 1.260, 95% confidence interval (CI) 1.157–1.374; *P* < 0.001], low muscle strength (OR 1.310, 95% CI 1.178–1.457; *P* < 0.001), and low physical performance (OR 1.216, 95% CI 1.104–1.339; *P* < 0.001). Furthermore, when NRI score was replaced by presence of medium- or high-risk, medium-/high-risk status retained a significant association with low muscle mass (OR 2.141, 95% CI 1.219–3.760; *P* = 0.008), low muscle strength (OR 7.665, 95% CI 3.438–17.091; *P* < 0.001), and low physical performance (OR 2.570, 95% CI 1.401–4.716; *P* = 0.002). Finally, when use of lipid-lowering medications was added to the multivariable logistic regression model, NRI score and medium-/high-risk status remained significantly associated with low muscle mass, low muscle strength, and low physical performance (Supplementary Table S1).

**Table 4:** Association of nutritional status with low muscle mass, low muscle strength, and low physical performance.

	**Low muscle mass**		**Low muscle strength**		**Low physical performance**	
	Adjusted*OR (95% CI)	***P*** **-Value**	Adjusted*OR (95% CI)	***P*** **-Value**	Adjusted*OR (95% CI)	***P*** **-Value**
NRI score	1.260 (1.157–1.374)	<0.001	1.310 (1.178–1.457)	<0.001	1.216 (1.104–1.339)	<0.001
Medium-/high-risk (ref. low-risk)	2.141 (1.219–3.760)	0.008	7.665 (3.438–17.091)	<0.001	2.570 (1.401–4.716)	0.002

* *Adjustments were made for age, gender, duration of hemodialysis, presence of diabetes mellitus, history of cerebrovascular disease, use of intravenous and/or oral vitamin D, hemoglobin, and CRP level*.

*OR, odds ratio; CI, confidence interval; NRI, nutritional risk index; CRP, C-reactive protein*.

### Associations of Nutritional Status With Sarcopenia and Severe Sarcopenia

NRI score was significantly higher in patients with sarcopenia [7.0 (4.0–9.0) vs. 4.0 (2.0–7.0), *P* < 0.001] as well as those with severe sarcopenia [8.0 (5.0–10.0) vs. 4.0 (3.0–8.0), *P* < 0.001], as compared to those without. Furthermore, the medium-/high-risk group demonstrated a significantly (*P* < 0.001) higher prevalence for sarcopenia and severe sarcopenia, as compared to the low-risk group (Table 2, Figure 1). To examine whether NRI score and medium-/high-risk based on NRI score were independently associated with sarcopenia and severe sarcopenia in the cohort, multivariable logistic regression analyses were performed (Table 5). NRI score was shown to be significantly associated with both sarcopenia (OR 1.255, 95% CI 1.143–1.377; *P* < 0.001) and severe sarcopenia (OR 1.257, 95% CI 1.122–1.407; *P* < 0.001). Furthermore, when NRI score was replaced by medium-/high-risk, a significant association with sarcopenia (OR 2.960, 95% CI 1.623–5.401; *P* < 0.001) as well as severe sarcopenia (OR 2.241, 95% CI 1.151–4.362; *P* = 0.018) was retained. Finally, when use of lipid-lowering medications was added to the multivariable logistic regression model, NRI score and medium-/high-risk status remained significantly associated with sarcopenia and severe sarcopenia (Supplementary Table S2).

**Table 5:** Association of nutritional status with sarcopenia and severe sarcopenia.

	**Sarcopenia**		**Severe sarcopenia**	
	Adjusted*OR (95% CI)	***P*** **-Value**	Adjusted*OR (95% CI)	***P*** **-Value**
NRI score	1.255 (1.143–1.377)	<0.001	1.257 (1.122–1.407)	<0.001
Medium-/high-risk (ref. low-risk)	2.960 (1.623–5.401)	<0.001	2.241 (1.151–4.362)	0.018

* *Adjustments were made for age, gender, duration of hemodialysis, presence of diabetes mellitus, history of cerebrovascular disease, use of intravenous and/or oral vitamin D, hemoglobin, and CRP level*.

*OR, odds ratio; CI, confidence interval; NRI, nutritional risk index; CRP, C-reactive protein*.

## Discussion

The present findings demonstrated that NRI score was significantly associated with muscle mass, muscle strength, and physical performance in the present Asian patients undergoing MHD. Furthermore, NRI score and medium-/high-risk status based on that score were significantly associated with sarcopenia and severe sarcopenia, as well as low muscle mass, low muscle strength, and low physical performance. These results suggest that NRI score, a nutritional screening tool, is useful for detection of sarcopenia and its related components. Furthermore, it is indicated that malnutrition contributes to sarcopenia and severe sarcopenia in MHD patients by reducing muscle mass and strength, as well as physical performance.

Some studies conducted previously have found significant associations of nutritional status with sarcopenia-related components, such as muscle mass (31), muscle strength (32, 33, 35, 36), and physical performance (34, 36, 37) in MHD patients using nutritional screening tools, including SGA and MIS, as well as geriatric nutritional risk index (GNRI). SGA and MIS, which include assessment of muscle mass, are considered to be validated clinical tools for screening nutritional status, though the results obtained are subjective and assessment is time consuming. While GNRI is an objective nutritional screening tool comprised of serum albumin level, current body weight, and ideal body weight, it does not include assessment of muscle mass. The present results demonstrated an association of nutritional status with all isolated sarcopenia-related components using the NRI, an objective nutritional screening tool that includes assessment of muscle mass, as reflected by serum creatinine concentration.

There are few reports regarding the relationship of nutritional status indicators with sarcopenia based on the combined components of sarcopenia in MHD patients. Giglio et al. found that SGA score was significantly lower in MHD subjects with sarcopenia as compared to without, based on the European Working Group on Sarcopenia in Older People (EWGSOP) criteria in a study conducted in South America (35). In Asia, one study demonstrated a significant association of MIS score with sarcopenia (38), while another found a non-significant association of modified versions of SGA score with sarcopenia (39). However, the number of subjects included in those studies was relatively few (<135 in each). Furthermore, in those investigations conducted in Asia, sarcopenia was defined based on the EWGSOP but not the AWGS criteria, in spite of cultural, lifestyle-related, and/or anthropometric differences between Asian and Western populations. In contrast, the results of the present study clearly demonstrate a significant association of nutritional status with sarcopenia/severe sarcopenia based on the combined components of sarcopenia as well as each individual component using regionally appropriate diagnostic criteria for sarcopenia in a relatively large number of patients undergoing MHD (*n* = 315).

Low dietary intake is known to be associated with low muscle and strength, and also low physical performance in older adults (40–42), while nutritional intervention in older patients was reported to increase those factors (43–45). Of importance, nutritional status was shown to predict development of sarcopenia in older adults (46), thus demonstrating that malnutrition contributes to sarcopenia in that population. In studies of MHD patients, nutritional intervention was found to result in increases in muscle mass, muscle strength, and physical performance (17–19). As a result, nutritional status is considered to have a significant effect on sarcopenia even in those patients (47). On the other hand, the association of nutritional status with sarcopenia and its components in MHD cases has not been fully investigated. In the present study, NRI score and medium- or high-risk status were significantly associated with sarcopenia and its related components. Thus, malnutrition may lead to sarcopenia or severe sarcopenia by reducing muscle mass, muscle strength, and physical performance in patients undergoing MHD. On the other hand, proactive nutritional intervention may prevent onset and progression of sarcopenia in MHD patients with medium-/high-risk based on the NRI, an objective and simple nutritional screening tool.

This study has some important limitations. First, due to the design, dietary intake, an important factor related to nutritional status as well as sarcopenia and its components (40–42), was not determined. Second, for nutritional status assessment, while NRI was used, other nutrition indicators, such as SGA or modified versions and MIS, were not determined. Thus, a large-scale longitudinal study of patients undergoing MHD that includes assessments of dietary intake and nutritional status by other tools in addition to NRI is needed to clarify the role of malnutrition in sarcopenia. Nevertheless, the present results clearly demonstrate that nutritional status is significantly associated with sarcopenia/severe sarcopenia based on the combined components of sarcopenia as well as each isolated component in MHD patients.

In conclusion, the present results showed that NRI score and medium- or high-risk status were significantly associated with sarcopenia and severe sarcopenia, as well as low muscle mass, low muscle strength, and low physical performance in patients undergoing MHD. It is thus suggested that malnutrition contributes to sarcopenia and severe sarcopenia in these patients by reducing muscle mass and strength, as well as physical performance.

## Data Availability Statement

The original contributions presented in the study are included in the article/Supplementary Materials, further inquiries can be directed to the corresponding author.

## Ethics Statement

The studies involving human participants were reviewed and approved by the Inoue Hospital Ethics Committee. The Ethics Committee waived the requirement of written informed consent for participation.

## Author Contributions

MK contributed to study design, interpretation, and writing of the manuscript. KM, MM, SM, MI, and AK contributed to study design and interpretation. YT contributed to study design, data analysis, and interpretation. TM and ME reviewed the manuscript. All authors have read and approved the final version of the manuscript.

## Conflict of Interest

The authors declare that the research was conducted in the absence of any commercial or financial relationships that could be construed as a potential conflict of interest.

## Publisher's Note

All claims expressed in this article are solely those of the authors and do not necessarily represent those of their affiliated organizations, or those of the publisher, the editors and the reviewers. Any product that may be evaluated in this article, or claim that may be made by its manufacturer, is not guaranteed or endorsed by the publisher.
