# Supplementary material for: Nutritional Status Association With Sarcopenia in Patients Undergoing Maintenance Hemodialysis Assessed by Nutritional Risk Index
Source: Front Nutr. 2022 May 13;9:896427. doi: 10.3389/fnut.2022.896427 (PMC9137182; doi:10.3389/fnut.2022.896427)
Supplement: Supplementary file 1 [file Table_1.DOCX]

**Supplemental Table S1. Association of nutritional status with low muscle mass, low muscle strength, and low physical performance independent of lipid-lowering medication use**

|  |  | **Low muscle mass** | |  | **Low muscle strength** | |  | **Low physical performance** | |
| --- | --- | --- | --- | --- | --- | --- | --- | --- | --- |
|  |  | **Adjusted* OR**  **(95% CI)** | **P** |  | **Adjusted* OR**  **(95% CI)** | **P** |  | **Adjusted* OR**  **(95% CI)** | **P** |
| Model 1 |  |  |  |  |  |  |  |  |  |
| NRI score |  | 1.261  (1.157-1.374) | <0.001 |  | 1.311  (1.178-1.460) | <0.001 |  | 1.216  (1.104-1.340) | <0.001 |
| Lipid-lowering medication use |  | 0.924  (0.513-1.664) | 0.793 |  | 1.480  (0.735-2.979) | 0.272 |  | 0.697  (0.358-1.354) | 0.287 |
| Model 2 |  |  |  |  |  |  |  |  |  |
| Medium-/high-risk  (ref. low-risk) |  | 2.151  (1.224-3.781) | 0.008 |  | 7.528  (3.368-16.828) | <0.001 |  | 2.655  (1.434-4.915) | 0.002 |
| Lipid-lowering medication use |  | 0.916  (0.522-1.609) | 0.760 |  | 1.301  (0.638-2.652) | 0.468 |  | 0.648  (0.336-1.250) | 0.196 |

*Adjustments were made for age, gender, duration of hemodialysis, presence of diabetes mellitus, history of cerebrovascular disease, use of intravenous and/or oral vitamin D, hemoglobin, and CRP level.

Abbreviations: OR, odds ratio; CI, confidence interval; NRI, nutritional risk index; CRP, C-reactive protein

**Supplemental Table S2. Association of nutritional status with sarcopenia and severe sarcopenia independent of lipid-lowering medication use**

|  |  | **Sarcopenia** | |  | **Severe sarcopenia** | |
| --- | --- | --- | --- | --- | --- | --- |
|  |  | **Adjusted* OR**  **(95% CI)** | **P** |  | **Adjusted* OR**  **(95% CI)** | **P** |
| Model 1 |  |  |  |  |  |  |
| NRI score |  | 1.255  (1.144-1.377) | <0.001 |  | 1.258  (1.122-1.409) | <0.001 |
| Lipid-lowering medication use |  | 1.265  (0.671-2.385) | 0.467 |  | 0.734  (0.340-1.586) | 0.432 |
| Model 2 |  |  |  |  |  |  |
| Medium-/high-risk  (ref. low-risk) |  | 2.935  (1.608-5.357) | <0.001 |  | 2.269  (1.159-4.444) | 0.017 |
| Lipid-lowering medication use |  | 1.175  (0.634-2.176) | 0.609 |  | 0.703  (0.332-1.491) | 0.358 |

*Adjustments were made for age, gender, duration of hemodialysis, presence of diabetes mellitus, history of cerebrovascular disease, use of intravenous and/or oral vitamin D, hemoglobin, and CRP level.

Abbreviations: OR, odds ratio; CI, confidence interval; NRI, nutritional risk index; CRP, C-reactive protein
